# An *in Vitro* Assay of hERG K^*+*^ Channel Potency for a New EGFR Inhibitor FHND004

**DOI:** 10.3389/fphar.2018.00577

**Published:** 2018-05-31

**Authors:** Tao Jin, Bingxue Hu, Shanshan Chen, Qiang Wang, Xue Dong, Yin Zhang, Yongqiang Zhu, Zhao Zhang

**Affiliations:** ^1^Jiangsu Key Laboratory for Molecular and Medical Biotechnology, College of Life Science, Nanjing Normal University, Nanjing, China; ^2^State Key Laboratory of Medical Neurobiology, School of Basic Medical Sciences and Institutes of Brain Science, and the Collaborative Innovation Center for Brain Science, Fudan University, Shanghai, China; ^3^Jiangsu Chia Tai Fenghai Pharmaceutical Co., Ltd., Nanjing, China

**Keywords:** FHND004, EGFR inhibitor, human *ether-à-go-go-*related gene, inhibitory effect, cardiac safety

## Abstract

FHND004 is a newly synthesized epidermal growth factor receptor (EGFR) inhibitor for the treatment of non-small cell lung cancer (NSCLC). The aim of the present study was to investigate the impacts of FHND004 on human *ether-à-go-go-*related gene (hERG) K^+^ channels and the molecular mechanisms underlying of its action. Whole-cell patch clamp recording was performed on wild type (WT), mutant hERG channels heterologously expressed in human embryonic kidney (HEK) 293 cells or *I*_Kr_ endogenously expressed in HL-1 cells, respectively. FHND004 inhibited hERG K^+^ currents in a concentration-dependent manner with IC_50_ values of 8.46 ± 0.33 μM in HEK293 cells and 7.52 ± 1.27 μM in HL-1 cells, respectively. However, the inhibitory potency of FHND004 on hERG channels was significantly less than its precursor AZD9291. FHND004-induced inhibition was state-dependent with a preference within open state, but did not alter other kinetics including activation, inactivation, and recovery from inactivation or deactivation. In addition, FHND004 exhibited more potent inhibitory effects on WT/A422T and WT/H562P-hERG, two known long QT syndrome (LQTS) associated *KCNH2* mutations, than WT alone. Mutations of the residues at pore regions (F656C, Y652A, S624A, and F557L) in hERG channels attenuated block effects of FHND004. Taken together, our results demonstrate the evidence that FHND004 is a less potent hERG blocker than its precursor AZD9291. There is, however, a need for caution in the potential use of FHND004 for treating NSCLC patients, especially in those with other concurrent triggering factors.

## Introduction

The human *ether-à-go-go*-related gene (hERG) encodes the pore-forming subunit of the rapid delayed rectifier K^+^ channels, which conduct the rapid delayed rectifier K^+^ current (referred as *I*_Kr_; Gutman et al., 2003) and play an essential role in the repolarization phase of the cardiac action potential. Inherited mutations of hERG can induce long QT syndrome (LQTS), a disorder of cardiac repolorization which predisposes affected individuals to life-threatening arrhythmias. In addition to inherited LQTS, a wide variety of drugs can also trigger ventricular arrhythmia and sudden death through blockade of hERG channels, such as Class III antiarrhythmics, antibiotics, antihistamines, antipsychotics, or anticancer agents (Mitcheson, 2008; Fradley and Moslehi, 2015). *In vitro* hERG assays therefore have become routine pre-clinical practice for most promising drug candidates, particularly for non-cardiac drugs (Food Drug Administration, HHS, 2005; Shah, 2005; Hancox et al., 2008; Han et al., 2011).

Epidermal growth factor receptor (EGFR) belongs to the receptor tyrosine kinase (RTK) super-family, which has a close relationship with the development of many human cancers (Hynes and Lane, 2005), especially in non-small cell lung cancer (NSCLC; Suzuki et al., 2005; Ellis et al., 2009; Rosell et al., 2009), thus making it an important anticancer target. For example, first- and second-generation EGFR tyrosine kinase inhibitors (EGFR-TKIs) erlotinib (Tarceva), gefitinib (Iressa), afatinib, and dacomitinib have been used in clinic for the treatment of different cancers, offering new therapeutic approaches and an effective long-term cancer therapy (Slamon et al., 2001; Perez et al., 2014). Unfortunately, acquired resistance to first- and second-generation EGFR-TKIs invariably develops within a median period of 9–13 months. Multiple mechanisms of acquired resistance have been identified, among which the mutation of the gatekeeper residue (T790M) is the most common one which was detected in 50% of clinically acquired drug-resistant patients (Sequist et al., 2011; Ohashi et al., 2013). Endeavors to overcome EGFR T790M-mediated resistance to first- and second-generation EGFR-TKIs have greatly promoted the development of third-generation EGFR TKIs, including WZ4002, CO1686, EGF816, and AZD9291(Cross et al., 2014; Janne et al., 2015; Sequist et al., 2015). Among them, AZD9291 is the only one recently approved by FDA for the treatment of advanced NSCLC patients with EGFR mutations and shows 200-fold selectivity for T790M/L858R double mutants over wild type (WT) EGFR (Cross et al., 2014; Jiang and Zhou, 2014). However, a few common adverse events of AZD9291, such as diarrhea, rash, and decreased appetite, have been reported in clinical trials. Moreover, the morbidity of diarrhea, rash, and cardiotoxicity increases with escalating doses of AZD9291 (Gao et al., 2016). To overcome these drawbacks, a novel compound FHND004 is synthesized based on the structure of AZD9291 with a modified pyrimidine ring and an expanded indole ring. Animal-based toxicity studies have shown that the adverse effects of FHND004 are significantly less intense than that of AZD9291, even with the increase of doses (Zhang et al., 2017).

Despite the advancements of EGFR-TKI-targeted anticancer drugs in the treatment of NSCLC, EGFR-TKI-associated cardiotoxicities including on-target toxicity and off-target toxicity are evident in some patients, such as symptomatic congestive heart failure (CHF), asymptomatic left ventricular (LV) dysfunction, or QT interval prolongation (Force et al., 2007; Chen et al., 2008). These unexpected cardiotoxicities are not wholly predicted by pre-clinical testing which contained animal toxicity studies, inhibition of hERG channels, and so on. As a fundamental component in evaluating the cardiotoxicities of an anticancer drug before being approved for clinical use, the inhibitory effects of hERG channels must be taken into consideration (Kerkela et al., 2006; Doherty et al., 2013). Although our previous results have evidenced that FHND004 exhibited a lower binding affinity to hERG channel than AZD9291 (Zhang et al., 2017), its specific mechanism underlying this inhibition remains to be investigated. Therefore, the aim of the present study is to integratively evaluate the impacts of FHND004 on hERG K^+^ channels.

## Materials and Methods

### Cell Culture and Transfection

The cardiac murine HL-1 cell line, a kind gift from William Claycomb (Louisiana State University, New Orleans), was grown in 0.02% fibronectin (Sigma, United States) precoated flasks and maintained in complete Claycomb medium (Sigma, United States) supplemented with 10% fetal bovine serum (FBS; Sigma, United States), 2 mmol/L L-glutamine (Invitrogen, United States), 100 mmol/L norepinephrine (Sigma, United States), 100 U/mL of penicillin (TransGen, China), and 100 μg/mL of streptomycin (TransGen, China) at 37°C in humidified 5% CO_2_ and 95% air.

Human embryonic kidney (HEK) 293 cells (Institute of Biochemistry and Cell Biology, Shanghai, China) were cultured in high glucose Dulbecco’s modified Eagle’s medium (DMEM; Gibco, United States) supplemented with 10% FBS (Wisent, Canada), 1% penicillin–streptomycin at 37°C in humidified 5% CO_2_ and 95% air.

The cgi-EGFP-hERG, cgi-EGFP-A422T-hERG, and cgi-EGFP-H562P-hERG plasmids were kindly gifted by Dr. Nipavan Chiamvimonvat (University of California, Davis, CA, United States). Transfections were performed using Lipofectamine^TM^ 2000 according to manufacturer’s instructions (Invitrogen, United States). EGFP-positive cells were used for current recording at least 36 h after transfection.

### Site-Directed Mutagenesis

Mutations of hERG channels (Y652A, F656C, S624A, and F557L) were constructed using the KFX-101 kit (Toyobo Co., Ltd., Japan) according to the manufacturer’s instructions. Each mutant was confirmed by sequencing and subsequently subcloned into the full-length cgi-EGFP vector. Plasmid vectors were then amplified in *Escherichia coli* DH5α and harvested for eukaryotic cell transfection as described above.

### Electrophysiological Recordings

Whole-cell patch clamp technique was employed to record *I*_hERG_ at room temperature (22 ± 1°C) using an AXON 200B amplifier through a digitizer (DigiData 1440A, Molecular Devices, United States) controlled by pClamp (v10.0) software. Cells were superfused with bath solution containing (mmol/L): NaCl 140, KCl 5.4, MgCl_2_ 1, HEPES 10, glucose 10, and CaCl_2_ 1 (pH 7.4). The pipette solution contained (mmol/L): KCl 140, MgCl_2_ 1, HEPES 10, K-ATP4, and EGTA 5 (pH 7.3). Borosilicate glass electrodes (World Precision Instruments Inc., United States) were pulled on a horizontal pipette puller (P-97, Sutter Instrument Co., United States) to resistances of 2–5 MΩ when filled with the pipette solution. The “pipette-to-bath” liquid junction potential calculated with this filling solution was about -2.7 mV. Because this value was small, no correction of membrane potential was made. Upon achieving a whole-cell configuration, series resistance (*R*_s_) was compensated by 85% to minimize voltage errors, and then pulses were applied until current amplitudes stabilized. Following the initial period of stable current recordings in bath solution, cells were superfused with the compounds at the concentrations as indicated in the results.

Specific voltage pulse protocols are described in Section “Results” and figure legends. The voltage dependence of current activation was determined by fitting the normalized tail current (*I*_tail_) vs. test potential (mV) to a Boltzmann function expressed by *I*_tail_*/I*_max_ = 1/(1+exp[(*V*_1/2_-*V*_m_)/k], where *V*_1/2_ is the voltage at which the current is half-activated and *k* is the slope factor. The time constants (tau) for inactivation (τ_inactivation_) and recovery from inactivation (τ_recovery_) were obtained by fitting a single-exponential equation: *y* = *A*_1_ × exp(-*x*/*t*_1_)+*y*_0_, respectively, where *t*_1_ represents the time constants. The time constants for deactivation (τ_deactivation_) were obtained by fitting a double-exponential equation: *y* = *A*_1_ × exp(-*x*/*t*_1_)+*A*_2_ × exp(-*x*/*t*_2_)+*y*_0_, where *t*_1_ represents the slow time constant (τ_slow_) and *t*_2_ represents the fast time constant (τ_fast_), respectively. Concentration responses for drug block of currents were fitted by the Logistic equation: *y* = *A*_2_+(*A*_1_-*A*_2_)/(1+(*x*/*x_0_*)^∧^*k*), where *A*_1_ and *A*_2_ represent the maximal and minimal inhibition, *x* refers to the working concentration of drugs, *x_0_* is the IC_50_ at which drugs produce a half-maximal inhibition of the *I*_hERG_, and *k* is the slope factor for the fit.

### Statistical Analyses

All data were expressed as the mean ± SEM. The significant differences of paired or unpaired data were determined by one-way ANOVA, followed by Tukey’s *post hoc* comparisons. All analyses were performed using Origin 8.6 software (Origin Lab Corporation, United States), and the value of *P* < 0.05 was designated as statistically significant.

## Results

### Concentration-Dependent Inhibition of FHND004 on *I*_hERG_

**FIGURE 1:**
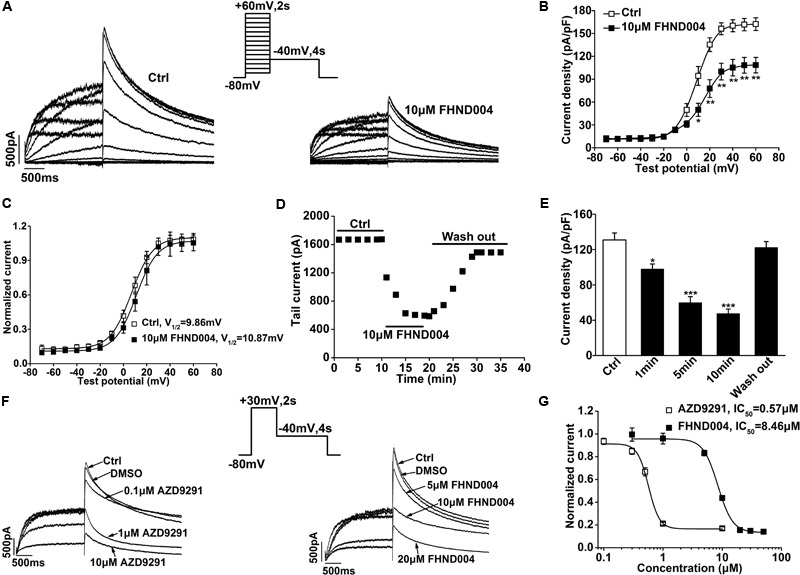
Effects of FHND004 on *I*_hERG_ recorded from HEK293 cells. **(A)** Families of *I*_hERG_ elicited by 2 s pulses to the potentials between –70 and +60 mV in 10 mV increments from a holding potential of –80 mV and then back to –40 mV for 4 s in the absence and presence of 10 μM FHND004. **(B)** *I*–*V* relationships for tail current in the absence and presence of 10 μM FHND004 (*n* = 8). **(C)** Steady-state activation curve fitted by a Boltzmann equation in the absence and presence of 10 μM FHND004, respectively. **(D)** Time course of FHND004-induced inhibition of hERG tail current from the same cells (*n* = 5). **(E)** The summarized peak current density in the absence and presence of 10 μM FHND004. **(F)** Peak current elicited by a 30 mV depolarization pulse for 2 s and then back to –40 mV for 4 s under various concentrations of AZD9291 and FHND004, respectively. **(G)** Concentration–response relationships fitted with logistic equation for block of hERG tail current by AZD9291 and FHND004, respectively (*n* ≥ 6 per data point). And the slope factor (*k*) for AZD9291 is 4.70 and *k* for FHND004 is 3.50. Panel **(G)** adapted from Zhang et al. (2017) with permission.

To investigate the effects of FHND004 on *I*_hERG_, whole-cell patch clamp recordings were performed on HEK293 cells heterologously expressing hERG channels. **Figure 1A** demonstrates the representative family of *I*_hERG_ upon a stepwise depolarization voltage ranging from -70 to +60 mV in 10 mV increments from holding potential of -80 mV, then to -40 mV (as shown in the inset) before and after the application of 10 μM FHND004. **Figure 1B** illustrates the current–voltage (*I*–*V*) relationships of hERG tail current before and after the application of 10 μM FHND004, demonstrating the significant inhibitory effects at +10 mV and beyond. And the steady-state activation of hERG current is depicted in **Figure 1C**; after being fitted to a Boltzmann distribution, the half-activation voltage (*V*_1/2_) increased slightly from 9.86 ± 0.54 to 10.87 ± 1.46 mV after the application of 10 μM FHND004, indicating that the steady-state activation was not affected by FHND004 (*P* > 0.05). As shown in **Figures 1D,E**, an inhibitory effect on tail currents was onset at 1 min and reached steady-state response at 5 min after drug application. FHND004-induced tail current decrease was recovered to 88.40 ± 0.46% by washing out, suggesting a reversible inhibition. To compare the inhibitory potency of FHND004 with its control AZD9291, the current recordings were performed with the protocol shown in the inset of **Figure 1F** in the presence of the testing compounds at different concentrations, respectively. Fitting the concentration–response relationships for block of hERG tail current with logistic equation gave the IC_50_ values of 0.57 ± 0.04 for AZD9291 and 8.46 ± 0.33 μM for FHND004 (*n* ≥ 6 per data point), respectively (**Figure 1G**). Obviously, the inhibitory effects of FHND004 on hERG channels were about 15-fold less than that of AZD9291.

**FIGURE 2:**
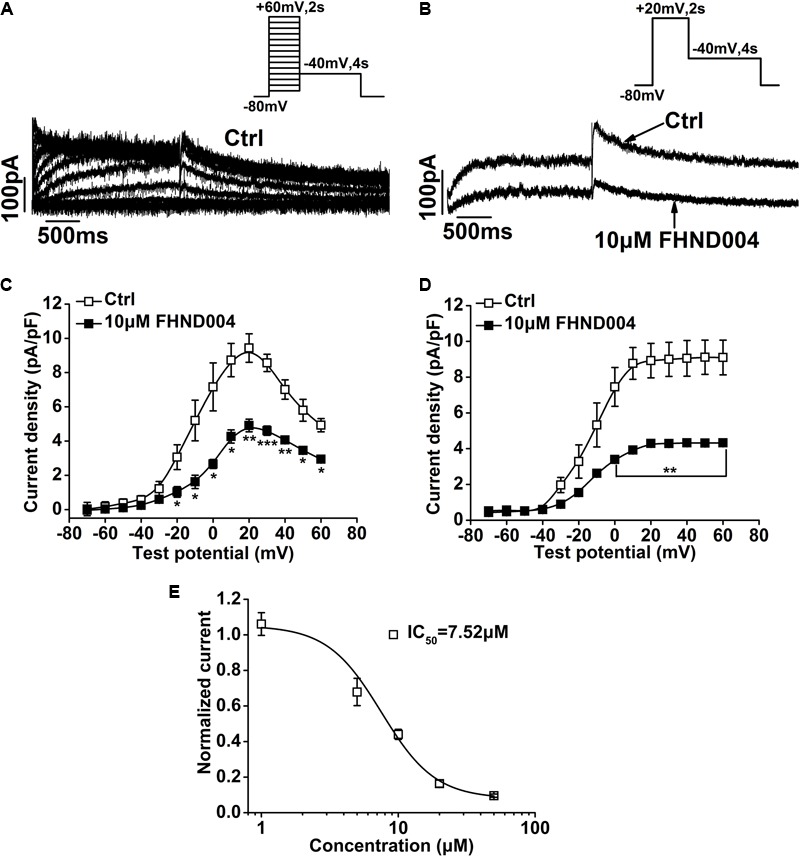
Effects of FHND004 on *I*_Kr_ recorded from HL-1 cells. **(A)** Families of hERG current elicited by the voltage protocol shown in the inset. **(B)** Peak current elicited by the voltage protocol shown in the inset in the absence and presence of 10 μM FHND004. **(C,D)** *I*–*V* relationships for step and tail currents in the absence and presence of 10 μM FHND004, respectively (*n* = 6). **(E)** Concentration–response relationships fitted with Logistic equation for block of *I*_kr_ tail current by FHND004 (*n* ≥ 4 per data point); *k* is 2.21 ± 0.67.

To further confirm the inhibitory effect of FHND004 on hERG channels, the cardiomyocyte-derived HL-1 cells (Claycomb et al., 1998; Caballero et al., 2017) were used to record *I*_Kr_ (**Figure 2A**). As it did in heterologous expression systems, FHND004 (10 μM) reduced both step and peak tail currents of *I*_Kr_ in HL-1 cells (**Figures 2B–D**). Additionally, concentration–response curve was plotted against each drug concentration, yielding an IC_50_ value of 7.52 ± 1.27 μM for block of tail current (**Figure 2E**; *n* > 4 per data point).

### Time-Dependent Inhibition of FHND004 on *I*_hERG_

**FIGURE 3:**
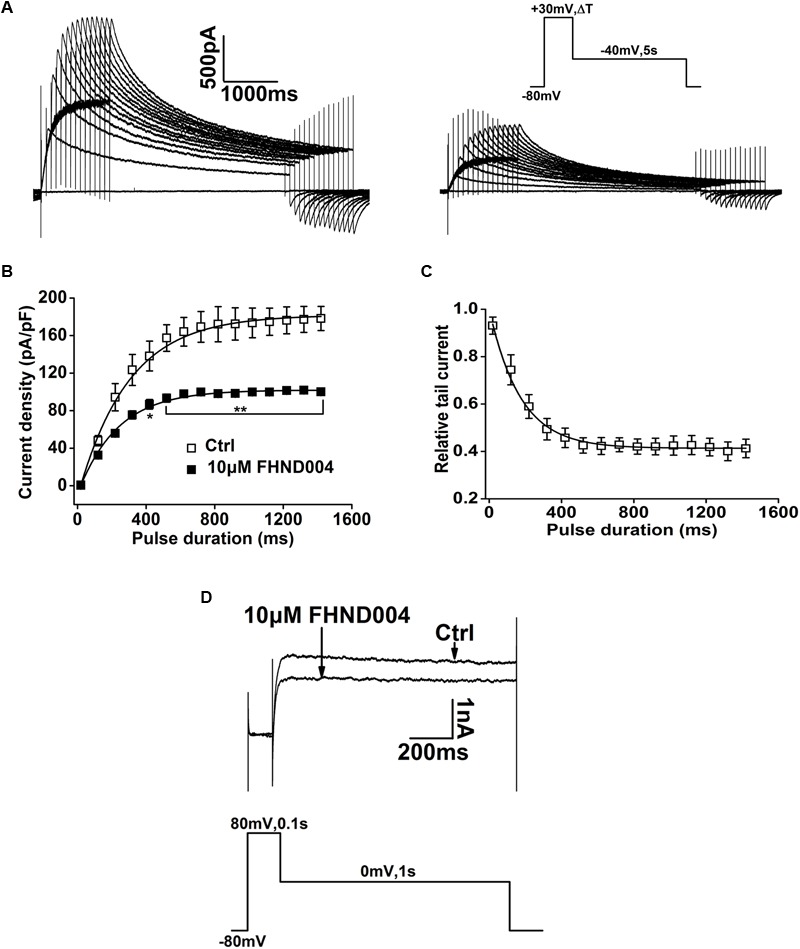
Open channel block of hERG by FHND004. **(A)** Representative current elicited by the “envelope of tails” protocol before and after application of 10 μM FHND004. Cells were held at a holding potential of -80 mV and pulsed to depolarizing voltage (+30 mV) for variable durations from 20 to 1420 ms in 100 ms increments. Envelope tail currents were recorded upon repolarization to -40 mV. **(B)** Mean plots of current density against development of depolarizing time fitted with single exponential equation before and after the application of 10 μM FHND004 (*n* = 6). **(C)** A plot of the onset of current block expressed as relative tail current [relative tail current = (currents with drug/currents without drug) × 100%]. The time-dependent decay in relative tail current was fitted to a mono-exponential equation (*n* = 6). **(D)** Representative current traces recorded before and after the addition of 10 μM FHND004, using the protocol shown in the inset (*n* = 5).

An “envelope of tails” protocol (as shown in the inset) was used to assess the development of inhibition on *I*_hERG_ by FHND004 over time during membrane depolarization (Cheng et al., 2012; Jie et al., 2017). The representative current traces before and after application of 10 μM FHND004 in **Figure 3A** showed that the tail current amplitude of *I*_hERG_ increased progressively as the length of the depolarizing step to +30 mV increased, with current amplitude relatively smaller in 10 μM FHND004 than that for the corresponding pulse protocol in control solution. **Figure 3B** illustrates the mean plots of current density against development of depolarizing time fitted with single exponential equation, exhibiting that FHND004 (10 μM) led to a minor decrease in the time constants of hERG channel activation (from 294.42 ± 13.22 ms for Ctrl to 261.48 ± 11.09 ms for drug application, *n* = 6). The currents with 10 μM FHND004 were expressed relative to control and fitted to a single exponential equation against pulse duration, which decayed in a pulse duration-dependent manner with a time constant of 172.26 ± 9.67 ms (**Figure 3C**, *n* = 6). **Figure 3D** (*n* = 5) shows the current recorded by using the classic protocol to study in details the possibility of hERG close-state block by drugs (Gualdani et al., 2015). The initial current during the step to +80 mV in the absence and presence of 10 μM FHND004 overlapped perfectly, suggesting minimal closed state block by FHND004. Therefore, these results illustrated an open channel block of hERG by FHND004.

### Effects of FHND004 on Kinetics of *I*_hERG_

**FIGURE 4:**
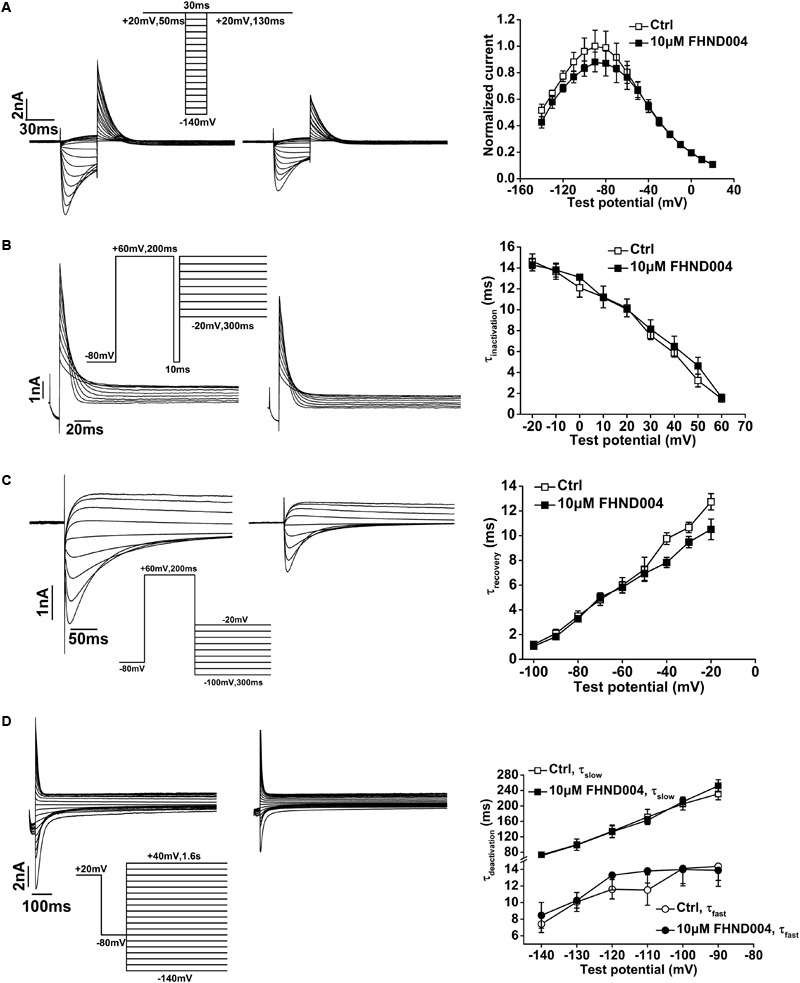
Effects of FHND004 on hERG channel kinetics. **(A)** Representative current traces of steady-state inactivation before and after the application of 10 μM FHND004 (left) with the protocol shown in the inset and *I*–*V* relationships of the outward inactivation current elicited during the second step to +20 mV obtained in the absence and presence of 10 μM FHND004 (right, *n* = 6). **(B)** Representative current traces of the time-course of hERG inactivation recorded by a three-pulse protocol before and after the application of 10 μM FHND004 (left) and inactivation time constant (tau) fitted by a single exponential equation (right, *n* = 5). **(C)** Representative current traces of recovery from inactivation before and after application of 10 μM FHND004 (left) and the time constant (tau) of recovery from the inactivation fitted by a single exponential equation (right, *n* = 6). **(D)** Representative current traces of deactivation before and after the application of 10 μM FHND004 (left) and the fast and slow time constants (tau) fitted by a double exponential equation (right, *n* = 7).

The effects of FHND004 on channel steady-state inactivation were assessed with the pulse protocol shown in insert in **Figure 4A**. After a 50 ms pulse to +20 mV to activate the channels, the membrane voltage was briefly stepped to different test potentials and then to +20 mV, evoking the current traces illustrated in **Figure 4A** before and after the application of 10 μM FHND004 (*n* = 6). Outward current amplitudes induced by second step to +20 mV were measured and normalized values were plotted as a function of the preceding test potentials. **Figure 4B** represents the normalized steady-state *I*–*V* inactivation curves for control and for FHND004 administration, respectively. Clearly, the steady-state inactivation of hERG channels was not affected by FHND004 (*P* > 0.05).

The effect of FHND004 on the onset of inactivation was investigated with a three-pulse protocol shown in inset of **Figure 4B**. A 200 ms prepulse from -80 to +60 mV was used to inactivate the channels, followed by a brief repolarizing pulse to -80 mV and then 300 ms test pulses from -20 to +60 mV in 10 mV increments were applied to elicit a large outward inactivating current. Representative currents obtained from both control and FHND004 treatment conditions are shown in **Figure 4B** (*n* = 5). Inactivation current traces were fitted by a single exponential function to extrapolate time constant values, indicating that the inactivation time constant was not affected by the presence of FHND004 (*P* > 0.05).

Recovery of hERG channels from inactivation was assessed with a protocol described by Zhou et al. (1998b). **Figure 4C** demonstrates the current of recovery from inactivation in the absence and presence of 10 μM FHND004 (*n* = 6). From a holding potential of -80 mV, a 200 ms prepulse to +60 mV was applied to inactivate the channels, and then, 300 ms test pulses from -100 to -20 mV in 10 mV increments were used to elicit the recovery current. Additionally, the time constant of recovery from inactivation fitted by a single exponential function was not affected by FHND004 (*P* > 0.05).

Deactivation of hERG channels was analyzed with a protocol shown in the inset of **Figure 4D** (Sharma et al., 2004). From a depolarization voltage of +20 mV, a short -80 mV stimulation was applied and then followed instantly by a series of 1.6 s test pulses from -140 to +40 mV in 10 mV increments to elicit the inward deactivating currents. Representative currents before and after the application of 10 μM FHND004 are demonstrated in **Figure 4D** (*n* = 7), along with the fast and slow time constants fitted by a double exponential function, showing that FHND004 treatment did not affect the deactivation time constants (*P* > 0.05).

### Alteration of hERG Channel Sensitivity to FHND004 Inhibition by Heterozygous Mutations in the *KCNH2* Gene

**FIGURE 5:**
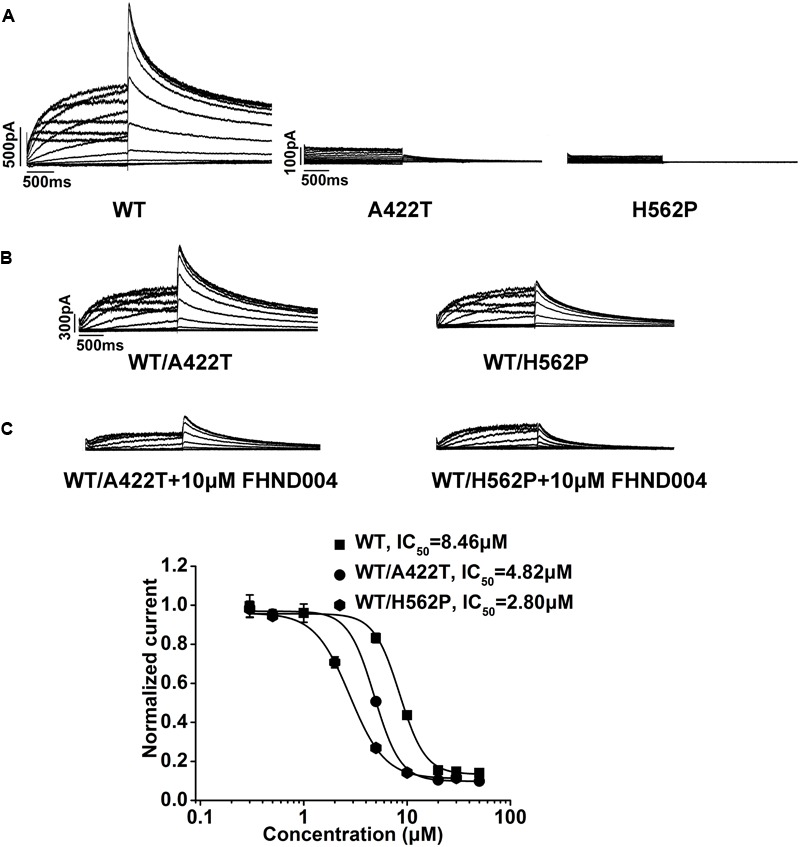
Effects of A422T and H562P mutations on FHND004-induced inhibition. **(A)** Representative current traces recorded from HEK293 cells expressing WT-hERG, A422T-hERG, and H562P-hERG K^+^ channels, respectively. **(B)** Representative current traces recorded from HEK293 cells expressing WT/A422T- and WT/H562P-hERG K^+^ channels in the absence of 10 μM FHND004. **(C)** Representative current traces recorded from HEK293 cells expressing WT/A422T- and WT/H562P-hERG K^+^ channels in the presence of 10 μM FHND004 and concentration–response relationships fitted with logistic equation for block of WT-, WT/A422T-, and WT/H562P-hERG tail currents by FHND004 with the same voltage protocol shown in **Figure 1E** (*n* ≥ 6 per data point), and the slope factors are 3.50, 3.45, and 2.55, respectively.

Multiple risk factors have been implicated in drug-induced QT prolongation, including electrolyte imbalance, bradycardia or congenital LQTS, and others. A422T and H562P are two LQT-associated *KCNH2* missense mutations that disrupt hERG protein trafficking to cell surface membrane (Zhou et al., 1998a; Delisle et al., 2004). To test the impacts of these heterozygous mutations on the hERG channel sensitivity to FHND004, the currents were recorded from the cells co-expressing WT/A422T-hERGand WT/H562P-hERG (1.5 μg for each) before and after the application of FHND004. As was previously reported by Guo et al. (2012), no currents were elicited from the cells expressing A422T or H562P mutant alone (**Figure 5A**, *n* = 5), while relative small currents were capable of being evoked from the cells heterozygously expressing WT/A422T-hERG or WT/H562P-hERG (**Figure 5B**) and the currents were further reduced in response to 10 μM FHND004 administration (**Figures 5B,C**, *n* = 6). More importantly, both mutations produced comparable shifts of the concentration response curves compared to WT channels for FHND004. As illustrated in **Figure 5C**, the IC_50_ values for FHND004 were dropped from 8.46 ± 0.33 to 4.82 ± 0.10 and 2.80 ± 0.06 μM, respectively, indicating that the inhibitory effect of FHND004 was approximately 1.8- and 3-fold more potent in WT/A422T-hERGand WT/H562P-hERG channels compared with WT hERG channels.

### Role of F656C, Y652A, S624A, and F557L Mutations on FHND004 Inhibition

**FIGURE 6:**
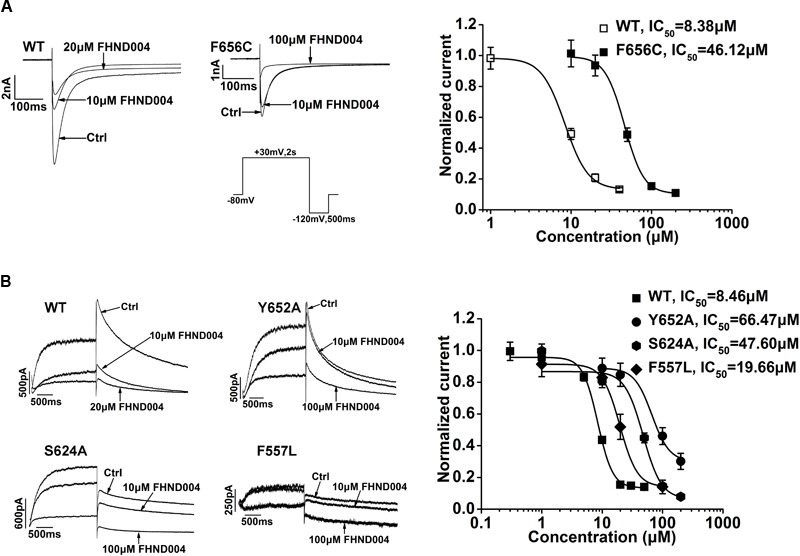
Molecular determinants of hERG channel block by FHND004. **(A)** Current traces of WT-hERG and F656C in high [K^+^]_e_ (94 mM) bath solution before and after the application of FHND004 with the protocol used to elicit the inward tail current shown in the inset and concentration–response relationships fitted with logistic equation for block of WT- and F656C-hERG inward tail current by FHND004 (*n* ≥ 5 per data point), and the slope factors are 3.00 and 3.61, respectively. **(B)** Current traces of WT-hERG, and Y652A, S624A, and F557L mutations before and after the application of FHND004 and concentration–response relationships fitted with logistic equation for block of WT- and Y652A-, S624A-, and F557L-hERG tail currents by FHND004 (*n* ≥ 6 per data point), and the slope factors are 3.50 ± 6.67, 3.00 ± 1.87, 2.86 ± 1.63, and 3.00 ± 1.39, respectively.

To understand the structure determinants for FHND004-induced blockade, critical amino acid replacement (i.e., F656C, Y652A, S624A, and F557L) at the hERG inner cavity was performed by site-directed mutagenesis. Corresponding results are shown in **Figure 6**, depicting the currents from WT-hERG and all mutant channels before and after the application of FHND004. Since F656C was comparatively poorly expressing (Mitcheson et al., 2000; Milnes et al., 2003; Kim et al., 2009), it was studied using a high [K^+^]_e_ (94 mM) bath solution, employing the protocol shown in the inset of **Figure 6A**. As expected, all the mutant channels, including F656C, Y652A, S624A, and F557L, showed less potent inhibition of FHND004, resulting in comparable rightward shifts of the concentration–response curves compared to WT channels (**Figures 6A,B**). The IC_50_ values described by logistic equation were 8.46 ± 0.33 μM for WT-hERG (8.38 ± 0.57 μM in 94 mM [K^+^]_e_), 46.12 ± 0.69 μM for F656C, 66.47 ± 0.83 μM for Y652A, 47.60 ± 0.66 μM for S624A, and 19.66 ± 0.78 μM for F557L, respectively. Therefore, these mutations located in hERG inner cavity attenuated FHND004 inhibition in the following order: Y652A > S624A > F656C > F557L, ranging from 7.9- to 2.3-fold decrease in the efficacy of FHND004.

## Discussion

FHND004 was a recently developed novel selective EGFR modulator based on the binding model of the marketed AZD9291 with T790M activity domain. Our previous study has evidenced that besides the potent activity in inhibiting EGFR T790M/L858R kinases and NSCLC cell growth *in vitro*, FHND004 exhibited significant *in vivo* antitumor efficacy and good pharmacokinetic properties with less toxicity than AZD9291 (Zhang et al., 2017). Although there is no available published clinical trial data, FHND004 might have the potential for long-term clinical use to treat patients with NSCLC in the future. Here, we took advantage of patch clamp technique to evaluate impacts of FHND004 on hERG channels in comparison with AZD9291.

In this study, our results elucidated that FHND004 was able to directly block hERG channels in a time- and concentration-dependent manner. However, FHND004 was a lower affinity blocker with IC_50_ value of 8.46 and 7.21 μM in non-cardiac cell line, HEK293, and in cardiac HL-1 cell line, respectively, which are about 15- and 13-fold greater than AZD9291. The maximum serum concentration (*C*_max_) of FHND004 and AZD9291 obtained from *in vivo* pharmacokinetic assay in male SD rats was both 0.22 μM (data not shown), giving rise to the margin of 38.5- and 2.5-folds between hERG IC_50_ and *C*_max_, respectively. Obviously, FHND004 has relative less potency to block hERG channels than AZD9291 with regard to safety margin. Also it is worth noticing that both drugs induced incomplete hERG block (about 85%) instead of 100% inhibition; we speculate that other than direct open channel block of AZD9291 and FHND004, maybe it involves more complicated mechanisms, including conformational alteration of hERG channels or formation of non-blocking intermediate complexes, which is worth further exploration in future study. Furthermore, dynamic analysis of channels demonstrated that the inhibition of FHND004 was associated with open channel without affecting the activation, deactivation, inactivation, and recovery from inactivation.

Besides the drugs themselves binding to the channels, clinical studies have identified multiple risk factors for the development of drug-associated LQTS, such as female, electrolyte disturbances, mutations in LQT genes, and others (Yang et al., 2002; Anderson et al., 2006; Mitcheson, 2008). Our studies elucidated that LQT2-associated A422T and H562P mutants rendered hERG channel more sensitive to the block effects of FHND004 comparing with WT channels. The molecular mechanisms, how the mutants influence drug affinity, are still poorly understood. Indeed, S5-located mutant of H562P was more sensitive to FHND004 than the S1-located A422T. Mutation at the H562 may disrupt these interactions, resulting in distortion of the channel pore or selectivity filter and consequently altered accessibility to FHND004. Nevertheless, LQT2 patients who harbor these mutations impairing K^+^ channel function may be specifically sensitive to FHND004 application and should be more cautious whenever it is possible to use. In addition, it should pay attention to that there existed the differences between our previous study which have done by Guo et al. (2012) and the present study regarding the dominant negative effect of heterozygously expressing WT/A422T-hERG. So, it needs to take into account whether the differences produce any impact on evaluation of changes in drug sensitivity.

It has been established that high affinity pharmacological inhibition of hERG channels is characterized by drug interactions with a binding site in the inner cavity involving multiple actions with pore-helical and S6 residues. For FHND004, mutation of the residue near the pore helix (S624; Mitcheson et al., 2000; Zhang et al., 2016) and two well-known residues in S6 (Y652 and F656; Sanguinetti and Mitcheson, 2005; Hong et al., 2010; Jie et al., 2017) significantly reduced channel sensitivity, suggesting that these residues act as the key binding determinants for FHND004, which also reduced the blockade of hERG by many other drugs, such as MK-499, dofetilide, ibutilide, cisapride, and terfenadine (Mitcheson et al., 2000; Perry et al., 2004; Kamiya et al., 2006, 2008). In contrast, mutation of F557 did not markedly alter channel sensitivity to FHND004, although it was considered as an essential residue in binding dofetilide and haloperidol (Saxena et al., 2016).

Besides a well-described cause of acquired LQTS on which much of the original *I*_Kr_ hypothesis was based, it has become increasingly evident that inhibition of intracellular signaling pathways, such as phosphoinositide 3-kinase (PI3K), is another cause of acquired LQTS (Qiu et al., 2016; Cohen et al., 2017). In this regard, in future, it is important to further investigate PI3K-denpendent effects of FHND004 that developed over time on multiple ion currents underlying repolarization and intracellular signaling pathways on native cardiomyocytes.

Inevitably, there is a major limitation which remains to be addressed in further studies; all the data in the present study were obtained from a recombinant system. Giving the complexity and largely non-selectivity of pharmacological compounds, their potential effects on multiple cardiac ion channels make it difficult to predict the combined effects on cardiac electrophysiology. On the other hand, drug-induced proarrhythmia can occur in the absence of hERG inhibition via direct inhibition of other repolarizing ion channels (Lane and Tinker, 2017). Therefore, it is worthwhile to further examine the effects of FHND004 on cardiomyocytes derived from human induced pluripotent stem cell (iPSC; Takasuna et al., 2017), or mature human ventricular tissue (Qu et al., 2017).

## Conclusion

Overall, FHND004 is able to directly block hERG channels at open state but shows less potency for inhibiting the channels than the marketed AZD9291. Nevertheless, special caution is still required whenever it is possible to use FHND004 for the treatment of NSCLC patients, in particular those patients with other risk factors, such as hereditary LQT syndromes, hyperkalemia, or elevated plasma levels of compounds induced by alteration in their pharmacokinetics, etc.

## Author Contributions

TJ, YoZ, and ZZ contributed to the conception and design of the study. TJ and BH carried out the electrophysiology experiments and analyzed the data. SC contributed to the molecular docking. YiZ, BH, and QW carried out cell culture and transfection. XD constructed the mutants. TJ and SC wrote the first draft of the manuscript. YoZ and ZZ revised the manuscript.

## Conflict of Interest Statement

SC is employed by company Jiangsu Chia Tai Fenghai Pharmaceutical Co., Ltd. The other authors declare that the research was conducted in the absence of any commercial or financial relationships that could be construed as a potential conflict of interest.
